# Incidence and predictors of intracranial bleeding after coronary artery bypass graft surgery

**DOI:** 10.3389/fcvm.2022.863590

**Published:** 2022-08-12

**Authors:** Ju Hyeon Kim, Pil Hyung Lee, Ho Jin Kim, Joon Bum Kim, Sojeong Park, Dae-Sung Kyoung, Soo-Jin Kang, Seung-Whan Lee, Young-Hak Kim, Cheol Whan Lee, Cheol Hyun Chung, Jae Won Lee, Seong-Wook Park

**Affiliations:** ^1^Department of Cardiology, Cardiovascular Center, Korea University Anam Hospital, Korea University College of Medicine, Seoul, South Korea; ^2^Division of Cardiology, Department of Internal Medicine, Asan Medical Center, University of Ulsan College of Medicine, Seoul, South Korea; ^3^Department of Thoracic and Cardiovascular Surgery, Asan Medical Center, University of Ulsan College of Medicine, Seoul, South Korea; ^4^Data Science Team, Hanmi Pharmaceutical Co., Ltd., Seoul, South Korea

**Keywords:** atherosclerosis, coronary artery bypass graft, intracranial bleeding, mortality, intracranial hemorrhage

## Abstract

**Background:**

There is a paucity of direct data on the incidence and predictors of intracranial bleeding (ICB) after coronary artery bypass graft surgery (CABG).

**Methods:**

The Korean National Health Insurance database was used to identify patients without prior ICB who underwent CABG. The outcomes of interest were the time-dependent incidence rates of ICB and the associated mortality.

**Results:**

Among 35,021 patients who underwent CABG between 2007 and 2018, 895 (2.6%) experienced an ICB during a median follow-up of 6.0 years. The 1-year cumulative incidence of ICB was 0.76%, with a relatively high incidence rate (9.93 cases per 1,000 person-years) within the first 1–30 days. Subsequent incidence rates showed a sharp decline until 3 years, followed by a steady decrease up to 10 years. The 1-year mortality rate after ICB was 38.1%, with most deaths occurring within 30 days (23.6%). The predictors of ICB after CABG were age ≥ 75 years, hypertension, pre-existing dementia, history of ischemic stroke or transient ischemic attack, and end-stage renal disease.

**Conclusions:**

In an unselected nationwide population undergoing CABG, the incidence of ICB was non-negligible and showed a relatively high incidence rate during the early postoperative period. Post-CABG ICB was associated with a high risk of premature death. Further research is needed to stratify high-risk patients and personalize therapeutic decisions for preventing ICB after CABG.

## Introduction

Coronary artery bypass graft surgery (CABG) offers a better survival rate than percutaneous coronary intervention (PCI) and is therefore the treatment of choice in patients with severe coronary artery disease (CAD). However, CABG entails a higher risk of stroke than PCI (1). Despite improvements in surgical techniques, equipment, and perioperative care, the incidence of post-CABG stroke has not significantly declined over the past decade (2–5). Hemorrhagic stroke, which is less frequent than ischemic stroke, is a devastating complication of CABG which carries a high risk of incapacitating disabilities and mortality (6). The mechanism responsible for intracranial bleeding (ICB) following CABG is different from that for ischemic stroke and may be related to modifiable factors such as prescription medicine. Therefore, both the in-hospital ICB and the ICB occurring after the early postoperative period should be considered clinically relevant.

Despite the importance of identifying the incidence pattern and risk factors for ICB in patients undergoing CABG, there is limited available information concerning this, including indirect data from drug trials or unselected stroke populations (7–10). A large-scale nationwide database would enable the analysis of every ICB event after CABG and allow more detailed analyses to guide preventive strategies in real-world clinical settings. Therefore, in the present study, we investigated the incidence, predictors, and prognostic impact of ICB in a nationwide population of patients undergoing CABG during a long-term follow-up period.

## Materials and methods

### Source of data

For data acquisition, we used nationwide cohort data from the National Health Insurance (NHI) database in South Korea, which is the single compulsory social insurance service that provides health coverage for all citizens. All healthcare providers are obligated to join the NHI system on a fee-for-service basis. The data include information on more than 50 million patients, covering 98% of the total population through this universal coverage system. All NHI claims are reviewed by a quasi-governmental organization [Health Insurance Review and Assessment Service (HIRA)] and are systematically classified and recorded in an independent computerized database (11, 12). From this claims database, a thorough follow-up for an individual is possible regardless of the region or hospital from which the medical service was provided. Thus, complete follow-up data is available until the last visit to any hospital or death. The database includes comprehensive information on healthcare services such as demographic findings, diagnoses, prescriptions, medical devices, and procedure records. Individual diagnoses are coded according to the International Classification of Diseases, 10th Revision (ICD-10). All prescribed medications were recorded with high accuracy and classified according to the chemical composition and dose of the drug. The institutional review board of Asan Medical Center (Seoul, South Korea) approved the study protocol and exempted the requirement for informed consent as the database used for the study consisted of anonymous, de-identified information.

### Study population

The study flow is presented in Supplementary Figure 1, and the definitions of diagnoses, procedures, and drugs are summarized in Supplementary Table 1. From the HIRA database, we identified patients aged ≥ 18 years who had undergone CABG between January 2007 and December 2018 to treat CAD. The screening period to assess the eligibility of each patient was set to at least 12 months before the index day. A diagnosis or procedure entered in the database remains permanently for an individual; therefore, we could ensure that the study included only those who underwent the first CABG by excluding patients in whom the database indicated a history of CAD and CABG during the screening period. CABG procedures were identified using the designated procedure codes (O1640–O1642, O1647–O1649, OA640–OA642, OA647–OA649). To create a more homogeneous risk population, patients who died on the day of CABG without a diagnosis of ICB, patients with any type of ICB before the index operation, or patients who underwent index CABG with concomitant valvular surgery were excluded.

### Study variables and endpoints

Clinical diagnoses that warranted CABG were categorized into either acute myocardial infarction or the others. We identified individual comorbid conditions such as hypertension, diabetes, dyslipidemia, history of heart failure, valvular heart disease, atrial fibrillation, peripheral artery disease, liver cirrhosis, end-stage renal disease requiring dialysis, cancer, dementia, and prior ischemic stroke or transient ischemic attack. The Charlson comorbidity index was used to measure the life expectancy (13). Further, it was determined whether CABG procedures were performed off-pump, and if they required mechanical circulatory support on the index day. Additionally, the use of antithrombotic medications [e.g., aspirin, P2Y12 inhibitors, vitamin K antagonist (VKA), and direct oral anticoagulants (DOACs)] was examined.

The primary endpoint of the study was the occurrence of non-traumatic ICB after the index CABG, considering that bleeding definitions consistently include ICB—regardless of the subtype—as the component for CABG-related major bleeding. The ICD-10 system classifies non-traumatic ICB as subarachnoid hemorrhage (I60), intraparenchymal hemorrhage (I61), subdural hemorrhage (I62.0), epidural hemorrhage (I62.1), and unspecified ICB (I62.9). To ensure that the event was a new one, ICB was qualified through both ICD-10 codes and brain imaging scans—either computed tomography or magnetic resonance imaging—during the hospitalization. Death was verified by all in- and outpatient claim records that indicated death. All claim data accrued until December 2020 were used, which allowed for at least 2 years of clinical follow-up for all study patients.

### Statistical analysis

Descriptive statistics for continuous variables are presented as median [interquartile range (IQR)] and were compared using the Wilcoxon rank-sum test. Categorical variables are presented as percentages and were tested using the chi-square test with the Yates continuity correction. Time-dependent rates of ICB and its subtypes were evaluated by incidence rates and are presented as the number of cases per 1,000 person-years. Mortality was estimated by the Kaplan–Meier method, and intergroup comparisons were assessed using the log-rank test. The multivariable Cox proportional hazards regression model was used to determine the predictors of ICB after CABG. The model included age (≥ 75 years), sex, acute myocardial infarction, diabetes, dyslipidemia, hypertension, congestive heart failure, valvular heart disease, atrial fibrillation, liver cirrhosis, end-stage renal disease (ESRD) requiring dialysis, peripheral arterial disease, cancer, pre-existing dementia, history of ischemic stroke or transient ischemic attack (TIA), off-pump coronary artery bypass graft surgery (OPCAB), use of mechanical circulatory support, use of VKA, use of DOACs, use of dual-antiplatelet therapy (DAPT), and use of statin. There was no relevant multicollinearity between variables assessed by the variance inflation factor values. Notably, occurrences of ICB on the day of index CABG were excluded for the multivariable Cox model. Factors associated with ICB on the day of index CABG were determined using binary logistic regression. In addition, the impact of ICB on mortality was examined using a time-dependent covariate analysis. All analyses included the entire dataset without any missing value. Statistical significance was defined at *P* < 0.05 for all two-sided tests. Data analyses were performed using SAS^®^ version 9.4 (SAS Institute Inc., Cary, NC, United States).

## Results

Overall, 6,806 patients underwent index CABG with concomitant valvular surgery, and were excluded from the final cohort (Supplementary Figure 1). A total of 35,021 patients met the eligibility criteria and were included in the current analysis. The baseline patient characteristics are presented in Table 1. The median age of the patients was 66 years, 74.0% were men, and 18.6% were diagnosed with acute myocardial infarction. Comorbid conditions included diabetes in 35.0% of the patients, atrial fibrillation in 5.0%, history of ischemic stroke in 13.8%, and end-stage renal disease in 3.5%. Additionally, OPCAB was performed in 60.4% of the patients, 78.0% of the patients were discharged with DAPT and only 7.6% received VKA.

**TABLE 1:** Baseline characteristics of patients with or without intracranial bleeding.

	Total (*N* = 35,021)	No ICB (*n* = 34,126)	ICB (*n* = 895)	*P*
Age, years	66 (58–72)	66 (58–72)	67 (60–73)	< 0.001
≥ 75 years	17.0	16.9	18.7	0.19
Male sex	74.0	74.0	70.9	0.04
Acute myocardial infarction	18.6	18.5	19.2	0.64
**Medical history**				
Diabetes	35.0	34.9	37.0	0.22
Dyslipidemia	67.1	67.2	65.0	0.19
Hypertension	83.6	83.5	87.6	0.001
Congestive heart failure	26.3	26.4	24.5	0.21
Valvular heart disease	1.8	1.8	2.5	0.19
Atrial fibrillation	5.0	4.9	6.0	0.16
Peripheral arterial disease	27.5	27.4	29.4	0.21
Liver cirrhosis	1.1	1.1	1.1	0.98
Chronic lung disease	36.6	36.6	37.5	0.59
ESRD requiring dialysis	3.5	3.4	6.1	< 0.001
History of ischemic stroke or TIA	15.8	15.7	21.5	< 0.001
Cancer	4.6	4.6	4.1	0.59
Pre-existing dementia	3.6	3.6	5.7	0.001
Charlson comorbidity index	3 (2–5)	3 (1–5)	3 (2–5)	< 0.001
Off-pump CABG	60.4	60.4	58.4	0.25
Use of mechanical circulatory support	3.3	3.3	2.6	0.26
**Medication at discharge**				
Single antiplatelet therapy	7.7	7.7	9.7	0.03
Dual antiplatelet therapy	78.0	78.1	74.6	0.02
Use of vitamin K antagonist	7.6	7.5	10.5	0.001
Use of direct oral anticoagulants*	1.1	1.1	0.9	0.68
Statin	86.5	86.6	82.8	0.001

Data are shown as median and interquartile range or percentage.

*Direct oral anticoagulants include dabigatran, rivaroxaban, apixaban, and edoxaban.

CABG, coronary artery bypass grafting surgery; ICB, intracranial bleeding; ESRD, end-stage renal disease; TIA, transient ischemic attack.

### Incidence of intracranial bleeding over time

**FIGURE 1:**
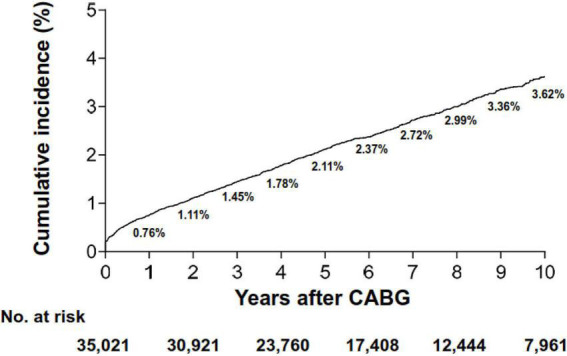
Cumulative incidence of intracranial bleeding. CABG, coronary artery bypass grafting surgery.

During a median follow-up duration of 6.0 years (IQR, 3.3–9.6) after CABG, a total of 895 patients were newly diagnosed with ICB, of which 530 (59.2%) cases had intraparenchymal hemorrhage. The risk of ICB accrued over time, with the rate of ICB at 30 days, 1 year, and 2 years being 0.30%, 0.76%, and 1.11%, respectively (Figure 1). The overall incidence rate was 3.98 cases per 1,000 person-years. Furthermore, the incidence rates had an early peak of 37.15 cases per 1,000 person-years within the first 30 days after the index surgery and showed a sharp decline until 3 years, followed by a steady decrease up to 10 years (Table 2). Notably, 73.3% (74/101) of early (< 30 days) ICB events occurred on the index day of CABG. The temporal trend of ICB occurrence was similar among different subtypes of ICB (Supplementary Table 2). In terms of the types of surgery, patients who underwent on-pump surgery showed a numerically higher cumulative incidence of ICB compared with those who underwent OPCAB (*P* = 0.09; Supplementary Figure 2). This difference was due to the different occurrence of ICB on the index day (49 cases in on-pump surgery vs. 25 cases in OPCAB).

**TABLE 2:** Incidence rates of intracranial bleeding according to time after index surgery.

Time after index surgery	No. at risk	No. of ICB cases	Incidence rate*
Overall	35,021	895	3.98 (3.73–4.25)
0–30 days	35,021	101	37.15 (30.57–45.15)
1–30 days	34,932	27	9.93 (6.81–14.48)
30 days to 1 year	33,718	154	4.72 (4.03–5.53)
1–2 years	31,895	111	1.75 (1.46–2.11)
2–3 years	30,919	99	1.07 (0.88–1.31)
3–4 years	27,213	87	0.80 (0.65–0.99)
4–5 years	23,759	75	0.63 (0.51–0.79)
5–6 years	20,427	51	0.42 (0.32–0.55)
6–7 years	17,380	57	0.47 (0.36–0.61)
7–8 years	14,743	39	0.33 (0.24–0.45)
8–9 years	12,440	42	0.38 (0.28–0.51)
9–10 years	10,087	24	0.24 (0.16–0.36)

*Reported as cases per 1,000 person-years (95% confidence interval).

CABG, coronary artery bypass grafting surgery; ICB, intracranial bleeding.

### Predictors of intracranial bleeding

Compared with the group that did not experience ICB, the ICB group had an older age, a higher proportion of female patients, and higher frequencies of hypertension, ESRD requiring dialysis, pre-existing dementia, and previous ischemic stroke or TIA (Table 1). Regarding the procedural characteristics, patients with ICB were more likely to receive on-pump surgery than those without ICB. Patients in the ICB group were more commonly treated with VKA and less commonly treated with statins.

The multivariable-adjusted independent predictors of ICB after CABG (excluding ICB on the day of index CABG; *n* = 74) included: age ≥ 75 years, hypertension, pre-existing dementia, history of ischemic stroke or TIA, and ESRD requiring dialysis (Table 3). Use of DAPT [hazard ratio (HR), 0.80; 95% confidence interval (CI), 0.63–1.00] and congestive heart failure (HR 0.84; 95% CI, 0.71–1.01) showed a borderline association with ICB, whereas the use of VKA, DOACs, or statins did not show significant associations with ICB. Factors associated with ICB on the index day (day 0) determined by logistic regression were on-pump surgery, CABG requiring mechanical circulatory support on the index day, and pre-existing dementia (Supplementary Table 3).

**TABLE 3:** Predictors of intracranial bleeding after index surgery (1 day–10 years).

Variables	Univariate	*P*	Multivariate	*P*
Age ≥ 75 years	1.60 (1.34–1.91)	< 0.001	1.51 (1.26–1.82)	< 0.001
Male sex	0.88 (0.76–1.02)	0.09	0.96 (0.83–1.12)	0.64
Acute myocardial infarction†	1.11 (0.93–1.32)	0.24	0.99 (0.82–1.19)	0.88
Diabetes	1.18 (1.02–1.36)	0.02	1.03 (0.88–1.20)	0.74
Dyslipidemia	1.00 (0.86–1.15)	0.94	0.89 (0.76–1.04)	0.13
Hypertension	1.49 (1.20–1.84)	< 0.001	1.26 (1.01–1.58)	0.04
Congestive heart failure	1.12 (0.96–1.32)	0.16	0.84 (0.71–1.01)	0.07
Valvular heart disease	1.54 (1.00–2.37)	0.05	1.32 (0.85–2.04)	0.22
Atrial fibrillation	1.46 (1.09–1.95)	0.01	1.14 (0.84–1.55)	0.39
Peripheral arterial disease	1.21 (1.04–1.41)	0.01	0.94 (0.79–1.11)	0.45
Liver cirrhosis	1.29 (0.67–2.48)	0.45	1.01 (0.52–1.97)	0.97
ESRD requiring dialysis	3.39 (2.56–4.50)	< 0.001	2.69 (1.97–3.68)	< 0.001
History of ischemic stroke or TIA	1.60 (1.36–1.89)	< 0.001	1.25 (1.04–1.50)	0.02
Cancer	1.07 (0.75–1.51)	0.72	0.79 (0.54–1.15)	0.21
Pre-existing dementia	2.11 (1.55–2.87)	< 0.001	1.40 (1.01–1.93)	0.04
On-pump surgery (vs. Off-pump CABG)	1.03 (0.89–1.18)	0.72	1.02 (0.89–1.18)	0.78
Use of mechanical circulatory support	1.28 (0.74–2.21)	0.38	1.23 (0.71–2.13)	0.46
Dual antiplatelet therapy	0.69 (0.59–0.82)	< 0.001	0.80 (0.63–1.00)	0.05
Use of vitamin K antagonist	1.59 (1.27–1.98)	< 0.001	1.20 (0.89–1.63)	0.24
Use of direct oral anticoagulants*	1.72 (0.85–3.46)	0.13	1.22 (0.59–2.55)	0.59
Use of statin	0.86 (0.71–1.03)	0.10	0.90 (0.74–1.08)	0.26

Values are hazard ratios (95% confidence interval).

†Hazard ratios are for patients with clinical presentation of acute myocardial infarction compared to those with angina.

*Direct oral anticoagulants include dabigatran, rivaroxaban, apixaban, and edoxaban.

CABG, coronary artery bypass grafting surgery; ESRD, end-stage renal dialysis; TIA, transient ischemic attack.

### Impact on mortality

**FIGURE 2:**
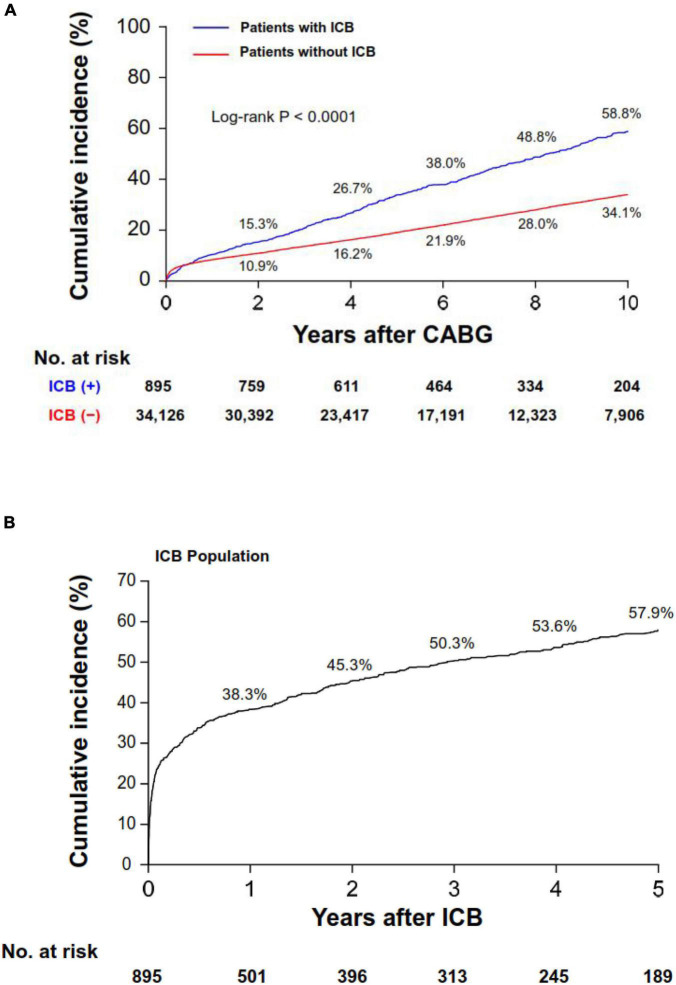
Cumulative incidence of all-cause mortality. Cumulative incidence curves for mortality in the overall population **(A)** and in the ICB population **(B)**. Note that panel **(B)** shows the cumulative incidence curve starting from the ICB event. ICB, intracranial bleeding; CABG, coronary artery bypass grafting surgery.

A total of 10,197 patients died during the follow-up period (1-year and 5-year mortality rates: 8.5% and 18.4%, respectively). Overall, patients who experienced ICB had higher crude rates of 5-year mortality compared with those who did not (33.7% vs. 19.1%, *P* < 0.001; Figure 2A), and this difference was evident after 30 days post-CABG. The cumulative incidence curve for mortality beginning from the time of the ICB event is shown in Figure 2B. The 1-year mortality rate after ICB was 38.3%, with most deaths occurring within 30 days (*n* = 211, mortality rate: 23.6%). Such a high rate of early death was observed regardless of the timing of the occurrence of ICB after index CABG (Supplementary Figure 3). In multivariable analyses, ICB was revealed as a strong independent predictor for all-cause mortality (HR, 4.32; 95% CI, 3.94–4.73; *P* < 0.001).

## Discussion

This current, contemporary study evaluated the incidence, predictors, and mortality impact of spontaneous ICB after CABG in an unbiased, real-world, nationwide patient population. The main findings of the analyses were that: (1) ICB complicated 2.6% of patients after CABG with an incidence rate of 4.0 cases per 1,000 person-years at a median of 6.0 years of follow-up; (2) ICB risk was the highest during the early postoperative period, and increased monotonically with a low incidence rate during the late period; (3) clinical risk factors were associated with the risk of ICB, whereas pharmacologic risk factors did not reach statistical significance; and (4) ICB was strongly associated with subsequent mortality, regardless of the timing of its occurrence after CABG.

Although ICB has been identified as the most severe form of bleeding following CABG by the Bleeding Academic Research Consortium definition, ICB in patients undergoing CABG has often been overlooked due to its low incidence (14). To our knowledge, no previous study has systemically assessed the real-world incidence of ICB after CABG. Previous drug trials and registry studies focusing on patients with acute coronary syndrome consistently reported a 1-year ICB incidence of 0.2–0.4% (15–20). However, CABG was performed in a minority of the patients (1–10%) in those studies, and their results do not fully represent our population of interest. A nationwide Taiwanese study evaluated the incidence of stroke after coronary or valve surgery and reported an incidence rate of 2.8% for in-hospital stroke among 49,919 patients who underwent CABG (21). Considering that 10.6% of all strokes were hemorrhagic strokes in the total cohort, the incidence rate of in-hospital ICB could be speculated to be 0.3%. Unlike these indirect data, our study directly provides the data on the actual incidence of ICB following CABG in a time-dependent manner, from the early postoperative period to 10-years postoperatively, in patients with diverse degrees of risk. We found that the 1-year rate of ICB was 0.76%, which is somewhat higher than that reported in previous studies on non-CABG CAD populations (22–27). Considering that several patient-related intrinsic risk factors were associated with ICB, a higher ICB rate in post-CABG patients than in the general CAD or PCI population seems reasonable. However, the high incidence rate in the early postoperative period highlights the potential influence of extrinsic factors associated with the operation, such as the types of surgery or the exposure time and dose of parenteral anticoagulation. As expected, the consequence of ICB in our cohort was critical in that nearly a quarter of patients died within 30 days. Furthermore, the difference in the mortality rates between those with and without ICB became more distinct after 30 days, suggesting a multifactorial relationship between ICB and mortality in the long term. Fortunately, the occurrence rate of ICB showed a steep decline and diminished to less than one case per 1,000 patient-years from the third year after index CABG.

The multivariable analysis of our nationwide data confirmed some of the previously suggested predictors of ICB. Firstly, patients aged ≥ 75 years and patients with a history of ischemic stroke or TIA were at higher risks for ICB, implying a higher probability of having a cerebrovascular pathology than their counterparts (24–27). Hypertension is also a well-established risk factor for ICB (26–28), and pre-existing dementia has been reported to be frequent in patients with ICB (29). In addition, the presence of ESRD was associated with more than a twofold increase in the risk of ICB (26). Considering that decreased renal function has been identified as a significant predictor for ICB in previous studies (26, 28), reduced clearance of antithrombotic drugs could be a plausible mechanism. We did not find anticoagulant, dual antiplatelet, or statin treatment to be associated with any increased risk of ICB. Since the majority (78.0%) were prescribed DAPT, mostly because of OPCAB (60.4%), the use of DAPT showed a borderline association with decreased hazard for ICB occurrence. However, oral anticoagulation after CABG is reserved for patients with atrial fibrillation, those with a history of venous thromboembolism, and those who undergo concurrent valvular surgery at the time of CABG. After excluding those who underwent index CABG with concomitant valvular surgery, neither VKA nor DOAC prescription reached statistical significance. Since only a small number of patients were prescribed either VKA (7.6%) or DOAC (1.1%) and the medication history was not a time dependent variable, these findings are inconclusive and are allowing only limited interpretation. Finally, it should be highlighted that these multivariable-adjusted independent predictors of ICB after CABG are very similar to ICB risk factors from the HAS-BLED score (30). Therefore, identifying those at high risk for post-CABG ICB based on intrinsic variables and making clinical decisions toward more balanced procedural and antithrombotic strategies would be a reasonable approach.

In South Korea, the case fatality rate of ICB in an unselected population was approximately 35% at 1 month (from the HIRA database) (31). In our analyses, the consequence of ICB was also severe, with the mortality at 30 days after ICB being 23.6%. The mortality risk showed no statistical difference according to the timing of the occurrence of ICB after index CABG. Given that the cumulative mortality curve continued to diverge for those with and without ICB, the impact of ICB on mortality in patients undergoing CABG is considerable during a long-term follow-up period.

### Study limitations

Although our study had several strengths, including its large size, recruitment of a nationwide CABG population, and identification of every ICB during a long-term follow-up period, it also has several limitations. First, due to the administrative nature of the NHI database, data on operative and laboratory variables—such as cardiopulmonary bypass time, heparin doses, platelet count, and preoperative brain imaging—were not available, which limited more detailed analyses of the potential predictors of ICB. Second, drug use was determined according to the history of prescription and not on actual adherence; moreover, the time in therapeutic range value in patients taking VKA was unavailable, which hindered robust assessment of the causal relationship between VKA use and ICB development. Third, the study design excluded patients with a history of either spontaneous or traumatic ICB, and this might have led to an underestimation of the ICB incidence rate after CABG. Fourth, the retrospective study design cannot address the ICB risk attributable to CABG. To determine the causality of CABG for ICB, patients who did not undergo CABG but had similar clinical risk profile are needed for competing risk analysis, which is not feasible in real-world settings. Finally, although our findings may have broad generalizability owing to the large size of the population, the study was restricted to Korean patients and specific circumstances (i.e., the first CABG without a previous diagnosis of CAD), potentially limiting the applicability of our findings to other ethnic groups or other high-risk patients.

## Conclusion

In a nationwide unselected population of patients undergoing CABG, the 1-year incidence of ICB was non-negligible (0.76%) with a particularly high incidence rate during the 30-day early postoperative period following CABG. Post-CABG ICB was associated with a high risk of premature death, regardless of the timing of its occurrence. Further studies are needed to stratify high-risk patients and personalize therapeutic decisions to prevent ICB following CABG.

## Data availability statement

The original contributions presented in the study are included in the article/Supplementary material, further inquiries can be directed to the corresponding author/s.

## Ethics statement

The studies involving human participants were reviewed and approved by the Institutional Review Board of Asan Medical Center (Seoul, South Korea). Written informed consent for participation was not required for this study in accordance with the national legislation and the institutional requirements.

## Author contributions

All authors listed have made a substantial, direct, and intellectual contribution to the work, and approved it for publication.
