# Supplementary material for: Incidence and predictors of intracranial bleeding after coronary artery bypass graft surgery
Source: Front Cardiovasc Med. 2022 Aug 12;9:863590. doi: 10.3389/fcvm.2022.863590 (PMC9411799; doi:10.3389/fcvm.2022.863590)
Supplement: Supplementary file 1 [file Data_Sheet_1.docx]

**Supplemental Material**

1. **Supplemental Tables**

**Table 1.** Definition of covariates and study outcomes

**Table 2**. Incidence rates of ICB according to ICB subtypes

**Table 3**. Factors associated with ICB on the day of index CABG

1. **Supplemental Figures**

**Figure 1**. Flow chart of the study population

**Figure 2.** Cumulative incidence of ICB according to Off-pump CABG

**Figure 3**. Cumulative incidence of mortality in patients who developed ICB at ≤ 1 year or > 1 year after CABG

**Table 1. Definition of covariates and study outcomes**

| **Diagnosis/Procedures/Drugs** | **Definition** |
| --- | --- |
| Coronary artery disease | I20–I25 |
| Coronary artery bypass grafting | O1640–O1642, O1647–O1649, OA640–OA642, OA647–OA649 |
| Intracranial bleeding | I60–I62 with brain imaging (CT or MRI) during the same hospitalization |
| Acute myocardial infarction | I21–I22 |
| Hypertension | I10–I13, I15 |
| Diabetes mellitus | E11–E14 and a minimum of one prescription of anti-diabetic drugs (sulfonylureas, metformin, α-glucosidase inhibitors, thiazolidinediones, meglitinides, dipeptidyl peptidase-4 inhibitors, sodium-glucose cotransporter 2 inhibitors, glucagon-like peptide-1 receptor agonist, and insulin) |
| Dyslipidemia | E78.0–E78.5 |
| Heart failure | I43, I50, I09.9, I11.0, I13.0, I13.2, I25.5, I42.0, I42.5–I42.9 |
| Valvular heart disease | I05–I09, I34–I39 |
| Atrial fibrillation | I48 |
| Liver cirrhosis | K70.3, K71.7, K74, K76.1, |
| Chronic lung disease | J40–J47, J60–J67, J68.4, J70.1, J70.3 |
| Renal disease | I13.1, N03, N05, N10–N19, Z49, Z94.0, Z99.2 |
| End-stage renal disease on dialysis | N18.5, Z49 and RID code V001, V003, V005 |
| Peripheral arterial disease | I70-I71, I73.1, I73.8, I73.9, I77.1, I79.0, I79.2, K55.1, K55.8, K55.9, Z95.8, Z95.9 |
| History of ischemic stroke | I63, I64 |
| History of transient ischemic attack | G45 |
| Dementia | F00, F01, F02, F03, G30, G311, F051 |
| Cancer | C00–C97 and RID code V027, V193, V194 |

CT, computed tomography; MRI, magnetic resonance imaging; RID, Rare Intractable Disease program.

**Table 2. Incidence rates of ICB according to ICB subtypes**

| Time after index CABG | No. at risk | No. of ICB cases | Incidence rate^*^ |
| --- | --- | --- | --- |
| **Intraparenchymal hemorrhage** | | | |
| Overall | 35,021 | 530 | 2.36 (2.16 – 2.56) |
| 0–30 days | 35,021 | 54 | 19.86 (15.21 – 25.94) |
| 1–30 days | 34,932 | 16 | 5.89 (3.61 – 9.61) |
| 30 days to 1 year | 33,718 | 101 | 3.10 (2.55 – 3.76) |
| 1–2 years | 31,895 | 66 | 1.04 (0.82 – 1.33) |
| 2–3 years | 30,919 | 53 | 0.57 (0.44 – 0.75) |
| 3–4 years | 27,213 | 60 | 0.55 (0.43 – 0.71) |
| 4–5 years | 23,759 | 40 | 0.34 (0.25 – 0.46) |
| 5–6 years | 20,427 | 31 | 0.25 (0.18 – 0.36) |
| 6–7 years | 17,380 | 32 | 0.26 (0.19 – 0.37) |
| 7–8 years | 14,743 | 23 | 0.20 (0.13 – 0.29) |
| 8–9 years | 12,440 | 26 | 0.23 (0.16 – 0.34) |
| 9–10 years | 10,087 | 11 | 0.11 (0.06 – 0.20) |
| **Subarachnoid hemorrhage** | | | |
| Overall | 35,021 | 164 | 0.73 (0.63 – 0.85) |
| 0–30 days | 35,021 | 18 | 6.62 (4.17 – 10.51) |
| 1–30 days | 34,932 | 6 | 2.21 (0.99 – 4.91) |
| 30 days to 1 year | 33,718 | 36 | 1.10 (0.80 – 1.53) |
| 1–2 years | 31,895 | 20 | 0.32 (0.20 – 0.49) |
| 2–3 years | 30,919 | 18 | 0.20 (0.12 – 0.31) |
| 3–4 years | 27,213 | 13 | 0.12 (0.07 – 0.21) |
| 4–5 years | 23,759 | 17 | 0.14 (0.09 – 0.23) |
| 5–6 years | 20,427 | 6 | 0.05 (0.02 – 0.11) |
| 6–7 years | 17,380 | 13 | 0.11 (0.06 – 0.18) |
| 7–8 years | 14,743 | 6 | 0.05 (0.02 – 0.11) |
| 8–9 years | 12,440 | 7 | 0.06 (0.03 – 0.13) |
| 9–10 years | 10,087 | 4 | 0.04 (0.01 – 0.11) |
| **Other** | | | |
| Overall | 35,021 | 272 | 1.21 (1.07 – 1.36) |
| 0–30 days | 35,021 | 35 | 12.88 (9.24 – 17.93) |
| 1–30 days | 34,932 | 7 | 2.58 (1.23 – 5.40) |
| 30 days to 1 year | 33,718 | 33 | 1.01 (0.72 – 1.42) |
| 1–2 years | 31,895 | 33 | 0.52 (0.37 – 0.73) |
| 2–3 years | 30,919 | 34 | 0.37 (0.26 – 0.52) |
| 3–4 years | 27,213 | 21 | 0.19 (0.13 – 0.30) |
| 4–5 years | 23,759 | 22 | 0.19 (0.12 – 0.28) |
| 5–6 years | 20,427 | 19 | 0.16 (0.10 – 0.24) |
| 6–7 years | 17,380 | 21 | 0.17 (0.11 – 0.27) |
| 7–8 years | 14,743 | 14 | 0.12 (0.07 – 0.20) |
| 8–9 years | 12,440 | 12 | 0.11 (0.06 – 0.19) |
| 9–10 years | 10,087 | 9 | 0.09 (0.05 – 0.17) |

^*^Reported as cases per 1000 person-years (95% confidence interval)

ICB, intracranial bleeding; CABG, coronary artery bypass grafting surgery.

**Table 3.** Factors associated with ICB on the day of index surgery (0 day)

| **Variables** | **OR (95% CI)** | ***P*** |
| --- | --- | --- |
| Age ≥ 75 years | 0.93 (0.50−1.71) | .80 |
| Male sex | 0.97 (0.57−1.63) | .89 |
| Acute myocardial infarction† | 1.40 (0.77−2.54) | .27 |
| Diabetes | 0.98 (0.57−1.68) | .93 |
| Dyslipidemia | 0.88 (0.53−1.47) | .63 |
| Hypertension | 1.01 (0.53−1.93) | .97 |
| Congestive heart failure | 1.12 (0.62−2.01) | .71 |
| Valvular heart disease | 0.66 (0.09−4.83) | .68 |
| Atrial fibrillation | 1.63 (0.69−3.87) | .27 |
| Peripheral arterial disease | 1.27 (0.72−2.22) | .41 |
| Liver cirrhosis | 1.39 (0.19−10.4) | .75 |
| ESRD requiring dialysis | 1.26 (0.36−4.43) | .72 |
| History of ischemic stroke or TIA | 0.83 (0.41−1.69) | .61 |
| Cancer | 1.55 (0.51−4.67) | .44 |
| Pre-existing dementia | 3.85 (1.68−8.83) | .001 |
| On-pump surgery (vs. Off-pump CABG) | 2.63 (1.60−4.30) | <.001 |
| Use of mechanical circulatory support | 3.08 (1.53−6.19) | .002 |

Values are odds ratios (95% confidence interval).

**Figure 1.** **Flow chart of the study population**


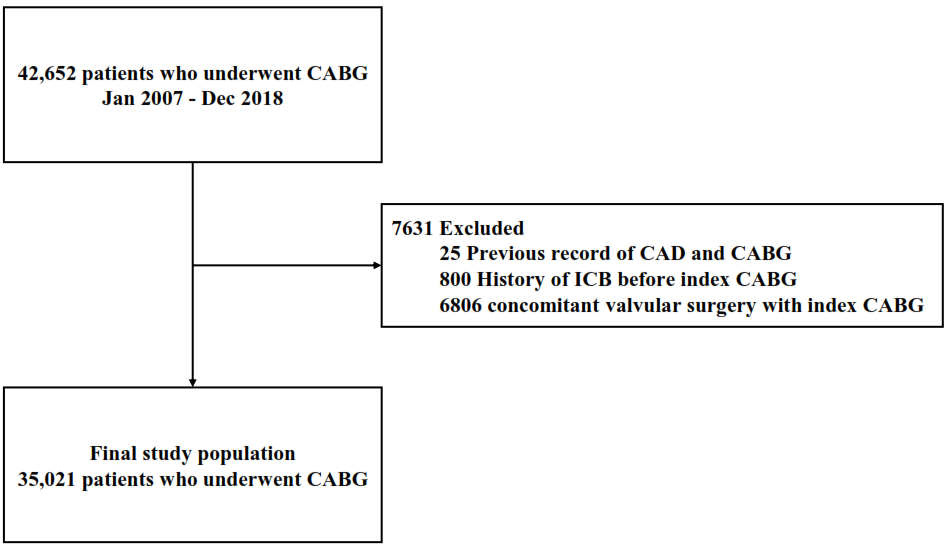


CABG, coronary artery bypass grafting; CAD, coronary artery disease; ICB, intracranial bleeding; PCI, percutaneous coronary intervention.

**Figure 2. Cumulative incidence of ICB according to Off-pump CABG**

**
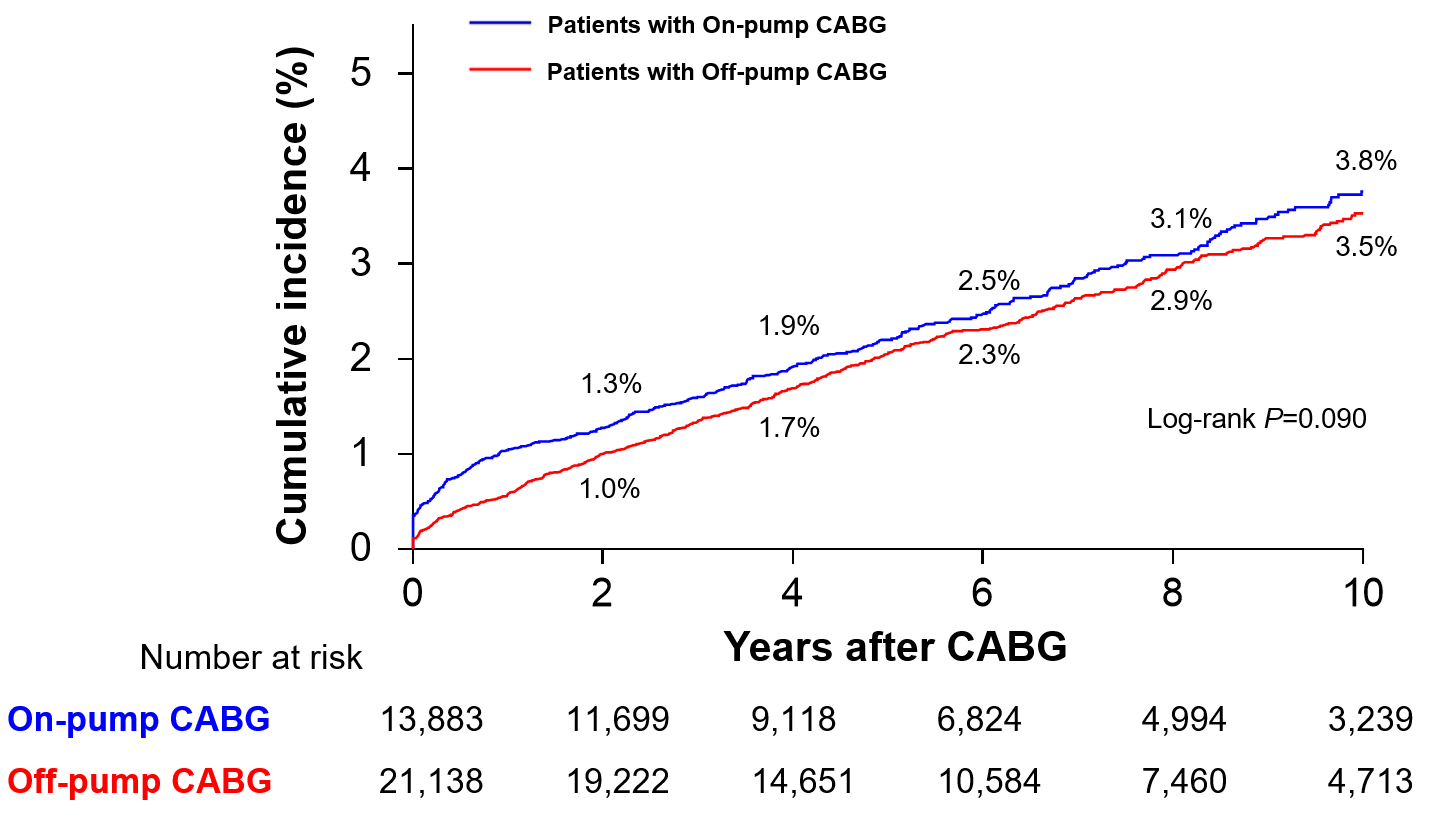
**

ICB, intracranial bleeding; CABG, coronary artery bypass grafting surgery; Valve Op, valvular surgery

**Figure 3. Cumulative incidence of mortality in patients who developed ICB at ≤ 1 year or > 1 year after CABG**


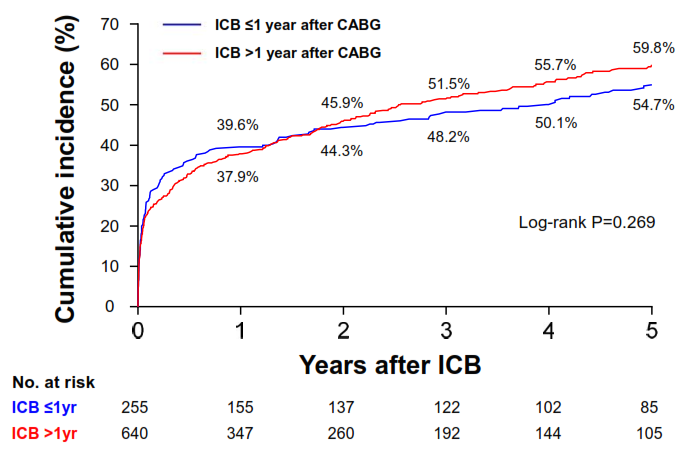


ICB, intracranial bleeding; CABG, coronary artery bypass grafting surgery.

|  |
| --- |
|  |
